# Promoting Beneficial and Inhibiting Undesirable Biofilm Formation with Mangrove Extracts

**DOI:** 10.3390/ijms20143549

**Published:** 2019-07-19

**Authors:** Yvana Glasenapp, Cristina Cattò, Federica Villa, Marco Saracchi, Francesca Cappitelli, Jutta Papenbrock

**Affiliations:** 1 Institute of Botany, Leibniz University Hannover, D-30419 Hannover, Germany; 2 Department of Food Environmental and Nutritional Sciences, Università degli Studi di Milano, 20133 Milano, Italy

**Keywords:** biofilm promotion, antibiofilm activity, mangrove extract, non-lethal concentration

## Abstract

The extracts of two mangrove species, *Bruguiera cylindrica* and *Laguncularia racemosa*, have been analyzed at sub-lethal concentrations for their potential to modulate biofilm cycles (i.e., adhesion, maturation, and detachment) on a bacterium, yeast, and filamentous fungus. Methanolic leaf extracts were also characterized, and MS/MS analysis has been used to identify the major compounds. In this study, we showed the following. (i) Adhesion was reduced up to 85.4% in all the models except for *E. coli*, where adhesion was promoted up to 5.10-fold. (ii) Both the sum and ratio of extracellular polysaccharides and proteins in mature biofilm were increased up to 2.5-fold and 2.6-fold in comparison to the negative control, respectively. Additionally, a shift toward a major production of exopolysaccharides was found coupled with a major production of both intracellular and extracellular reactive oxygen species. (iii) Lastly, detachment was generally promoted. In general, the *L. racemosa* extract had a higher bioactivity at lower concentrations than the *B. cylindrica* extract. Overall, our data showed a reduction in cells/conidia adhesion under *B. cylindrica* and *L. racemosa* exposure, followed by an increase of exopolysaccharides during biofilm maturation and a variable effect on biofilm dispersal. In conclusion, extracts either inhibited or enhanced biofilm development, and this effect depended on both the microbial taxon and biofilm formation step.

## 1. Introduction

Microorganisms, including fungi, adhere to surfaces and form complex and heterogeneous microbial communities called biofilms. It is well known that the formation of biofilms is undesirable in some systems, including water supply and food plants [1,2], heat exchangers and cooling towers, corrodible metal surfaces [3,4,5], and biomedical devices [6]. In contrast, the employment of biofilms in other environments, such as for bioremediation, in wastewater treatment plants [7,8], in other industrial settings [9], and for microbial fuel cells [10] is remarkably beneficial. In the former case, research has aimed at preventing adhesion, reducing maturation, and favoring detachment. In the latter case, the enhancement of initial adhesion to a substratum and the subsequent formation of robust mature biofilms are important goals to achieve.

Despite their beneficial roles in many fields, biofilms are mainly associated with negative impacts. In this direction, antimicrobial agents aimed at killing microorganisms have been extensively employed to control biofilm growth. However, since sessile cells can be up to 1000 times more resistant to antimicrobial agents than their planktonic counterpart, controlling their growth could be significantly challenging once they are formed [11]. Moreover, the emergence of resistant microorganisms is favored by the inappropriate use of antibiotics, as well as unsatisfactory infection prevention and control, posing serious concerns for public health and food security worldwide [12,13]. Thus, in recent years, researchers have focused their attention on alternative, safe, cost-effective and more eco-sustainable antibiofilm strategies employing non-toxic and biodegradable solutions. On the contrary, agents promoting biofilm development have been less investigated [14].

Plants have a long history of being a source of interesting compounds. Also at present, plant metabolites are the origin of newly developed drugs next to other natural sources and synthetic compounds [15]. These compounds are secondary metabolites that are synthesized by the plant to cope with environmental stress or for reproductive purposes. In most cases, these molecules display suitable environmental fate parameters such as high water solubility, low log P and bioaccumulation in the biological systems, and no ecotoxicity, which make them potential safe antibiofilm or biofilm-promoting agents [16,17]. Mangroves are trees and shrubs growing along tropical coasts with direct contact to seawater, which is an environment with many biotic and abiotic stress factors. In response to their surroundings, mangroves produce diverse secondary metabolites, including flavonoids, tannins, alkaloids, and terpenes [18]. They are used in ethnobotany for the treatment of several diseases, including infections, and also as pesticides [19].

In this study, mangrove extracts were employed to study their effects on biofilm formation (i.e., adhesion, maturation, and detachment) of three microbial models: a bacterium (*Escherichia coli*), a yeast (*Candida albicans*), and a filamentous fungus (*Fusarium oxysporum*). An advantage of using the extracts is their putative additive and synergistic activity, as they can act on multiple targets, which is an interesting strategy when dealing with the complex biofilm formation phenomenon, as several pathways are involved [20]. Mangroves were chosen as they have already been proven to be promising in interfering with planktonic and sessile bacteria and fungi [21,22].

Many researchers have proved that several phytochemicals show activity on the adhesion of different microorganisms. Lin et al. [23] showed the inhibitory effect of 1,2,3,4,6-penta-O-galloly-beta-D-glucopyranose on *Staphylococcus aureus* biofilm formation. In the study by Das et al. [24], the inhibition of *Pseudomonas aeruginosa* adhesion was achieved by using vitexin together with azithromycin and gentamicin. The effects of myricetin derivatives were studied on biofilms formed by many microorganisms. Lopes et al. [25] reported that myricetin, together with hesperetin and phloretin, inhibited *Staphylococcus aureus* RN4220 biofilm formation by more than 70%, and lower biofilm formation inhibition was recorded against *S. aureus* SA1199B. Similarly, Arita-Morioka et al. [26] examined activities of myricetin derivatives to reduce adhesion by *E. coli* K-12. In the case of *P. aeruginosa* PAO1, Pejin et al. [27] suggested the delphinidin scaffold for the design of the new potent antibiofilm agents. The inhibitory activity of tea catechin epigallocatechin-3-gallate against biofilms by *Streptococcus mutans* and a *Lactobacillus* sp. was observed by Wu et al. [28]. De Vita et al. [29] reported that caffeic acid and several acid derivatives were able to inhibit *C. albicans* adhesion.

Although in several papers the inhibition of biofilm formation has been discussed and some manuscripts reported an enhancement of biofilm formation upon exposure to phytochemicals [24,25,29,30], no manuscripts have reported biofilm inhibition and promotion in the same study. To the best of our knowledge, this work shows for the first time that the effects of non-lethal concentrations of plant extracts are dependent on the microbial strain and biofilm formation step.

Here, we compared biofilm features in response to phytochemicals on prokaryotic, yeast-like, and filamentous fungal cells. Notably, so far, a number of natural compounds have been proposed for controlling bacterial and yeast fungal biofilms, probably because these microorganisms have been recognized to have a large impact on human health and the economy. In contrast, only few studies have dealt with safe options for controlling filamentous fungal biofilm. In the past, they were generally assumed not to form biofilms, probably because they do not completely fit biofilm definitions based on bacterial and yeast models [31].

## 2. Results

### 2.1. Phytochemical Analysis of the Extracts

#### Total Phenol Content (TPC), Total Flavonoid Content (TFC) and Oxygen Radical Absorbance Capacity (ORAC)

**Figure 1:**
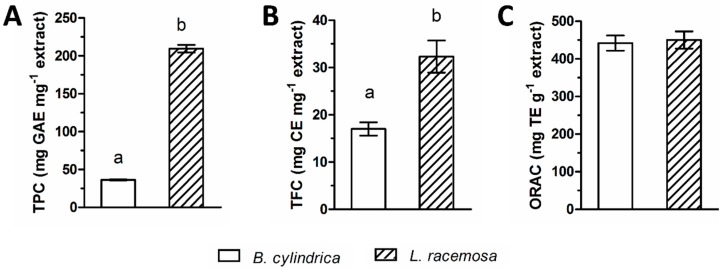
Crude methanolic extracts were analyzed for total phenols in mg gallic acid equivalent (GAE) per mg extract (**A**), total flavonoids in mg catechin equivalent (CE) per mg extract (**B**), and oxygen radical absorbance capacity (ORAC) in mg Trolox equivalents (TE) per g extract (**C**). Data represent the mean ± SDs and different superscript letters indicate statistically significant differences (Tukey’s HSD, *p* ≤ 0.05) between the means of three independent measurements.

Both mangrove species contained phenolic acids and flavonoids in the methanolic extract from fresh leaf material (Figure 1). The concentration of flavonoids and phenolic acids was higher in *Lagunclaria racemosa* (32.31 ± 3.41 µg catechin equivalent (CE) and 209.55 ± 4.97 µg gallic acid equivalent (GAE)/mg extract) compared to *Bruguiera cylindrica* leaf extract (17.00 ± 1.43 µg CE and 36.39 ± 0.59 GAE/mg extract). The ORAC value was similar for both species with 450.20 ± 23.09 mg Trolox equivalents (TE)/g extract for *L. racemosa* and 441.99 ± 20.23 mg TE/g extract for *B. cylindrica*.

### 2.2. LC-MS Analysis of Secondary Metabolites

Crude methanol (MetOH) extracts of *B. cylindrica* and *L. racemosa* leaves were further analyzed for their secondary metabolite composition by LC-MS (Table 1 and Figure S1). Mass signals in the range of 100 to 800 Da in ESI showed varying signals for both species. MS and MS/MS data obtained from the analysis were compared to publicly available databases. In both extracts, the sugar galactinol was identified. Another sugar, mannitol, was present in *L. racemosa* extract. Not all the peaks in the extract could be identified with the databases used. Of the identified signals, two belonged to the group of phenolic acids in the *B. cylindrica* extract, namely caffeic acid and 1-O-b-D-glucopyranosyl sinapate modified. Hirsutine, an indole alkaloid, was present as well, in addition to the flavones homoorientin, vitexin-2″-O-rhamnoside, and one of its isomeric forms. In the extract from *L. racemosa* leaves, the flavonoids (–)-epigallocatechin, epigallocatechin 3,5-digallate, delphinidin, and myricetin were detected, in addition to the flavones robinetin trimethyl ether, myricitrin, and two of its glycosidic forms. The signal of methyl gallate was likely a breakdown product from epigallocatechin 3,5-digallate. The tannin casuarinin was identified by the presence of the double-charged ion at m/z 467.03, and its fragments were compared to the data measured by Glasenapp et al. [21].

### 2.3. Mangrove Extracts do not Have Toxic Effects

#### 2.3.1. Mangrove Extracts do not Have a Biocidal Effect

*E. coli*, *C. albicans*, and *F. oxysporum* growth were undisturbed in the presence of both mangrove extracts at all the concentrations tested, with no discernible inhibition halo around the disks. Both the negative controls gave the same negative result, indicating that the addition of 2% MetOH to each extract did not kill microorganisms. On the contrary, a visible inhibition halo was present around the disk imbibed with the positive controls (Figure S2).

#### 2.3.2. Mangrove Extracts do not Modulate Planktonic Growth

The ability of mangrove extracts to modulate planktonic growth was tested by monitoring the microbial growth over time. The grow curves obtained for *E. coli*, *C. albicans*, and *F. oxysporum* in the presence of *B. cylindrica* and *L. racemosa* extracts at all the concentrations tested are reported in Figure S3. Furthermore, the interpolation of obtained data with the Gompertz growth model provided kinetic parameters is reported in Table S1. Experiments did not show differences in the duration of lag phase (λ), maximum specific growth rate (μ_m_), and maximum growth (YM) when all the microbial models where grown with or without both mangrove extracts. Therefore, mangrove extracts did not modulate microbial growth up to 1000 mg/L.

No differences were observed between the phosphate buffer saline (PBS) and 2% MetOH negative controls for each of the microorganisms tested.

#### 2.3.3. Mangrove Extracts do not Affect *F. oxysporum* Conidia Germination

Experiments showed that after 21 h of incubation with or without *B. cylindrica* and *L. racemosa* extracts, 100% of conidia were germinated. No differences were observed between the PBS and 2% MetOH negative controls.

### 2.4. Mangrove Extracts are not a Carbon and Energy Source

Both *B. cylindrica* and *L. racemosa* were not a carbon and energy source for *E. coli*, *C. albicans*, and *F. oxysporum*, as no microbial growth was detectable either when the two extracts were present as the sole carbon and energy source or in the control plates with no organic matter. On the contrary, in the control with sucrose, microbial growth was observed (Figure S4).

### 2.5. Mangrove Extracts Affect Cells/Conidia Adhesion

**Figure 2:**
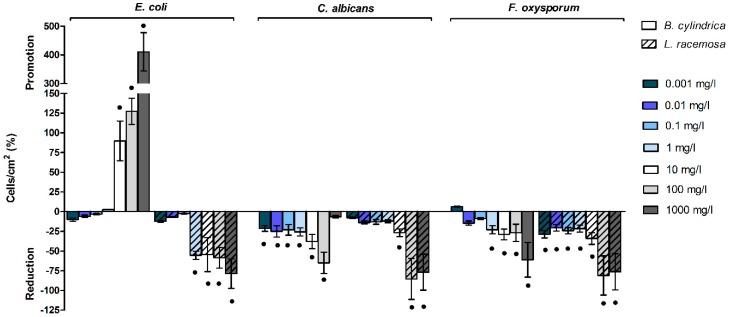
Cells/conidia adhesion in the presence of *B. cylindrica* and *L. racemosa* extracts. Percentage differences with respect to the negative control plus 2% MetOH are reported. Black dots indicate statistically significant differences with the 2% MetOH negative control.

The effects of mangrove extract on cells or conidia adhesion and the percentage differences with respect to negative controls with 2% MetOH are reported in Figure 2 and Table S2.

Mangrove extracts showed an opposite effect on *E. coli* cells’ adhesion. Above 10 mg/L, *B. cylindrica* promoted microbial adhesion, up to 5.10 ± 0.66-fold in comparison to the 2% MetOH control sample. On the contrary, above 1 mg/L, *L. racemosa* abated the number of adhered cells up to the 78.7 ± 18.8% at the highest concentration tested.

Both mangrove extracts decreased *C. albicans* cells and *F. oxysporum* conidia adhesion, with a major effect of the *L. racemosa* extract in comparison to the *B. cylindrica* extract. Indeed, *B. cylindrica* displayed low activity between 0.001–1 mg/L, moderate activity at 10 mg/L, and excellent activity at 100 mg/L against *C. albicans* adhesion, whereas at 1000 mg/L, no effect was observed. *L. racemosa* slightly reduced *C. albicans* adhesion at 10 mg/L and severely decreased the number of adhered cells at 100 mg/L and 1000 mg/L.

As regards *F. oxysporum*, the *B. cylindrica* extract displayed low activity from 1 to 100 mg/L and excellent activity at 1000 mg/L. A major effect was shown in the presence of the *L. racemosa* extract, which exhibited low performances from 0.001 mg/L to 1 mg/L, moderate effect at 10 mg/L, and optimal anti-adhesion effects at 100 mg/L and 1000 mg/L.

No differences were shown between the PBS and 2% MetOH negative controls for each of the microorganisms tested.

### 2.6. Mangrove Extract Modulate Biofilm Maturation

#### 2.6.1. Mangrove Extracts Affect Cellular Biomass Abundant

**Figure 3:**
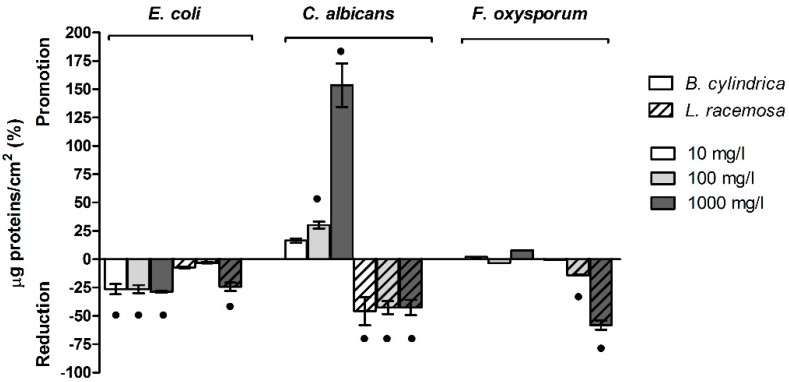
Cellular biomass within the biofilm in the presence of *B. cylindrica* and *L. racemosa* extracts. Percentage differences with respect to the negative control plus 2% MetOH are reported. Black dots indicate statistically significant differences with the 2% MetOH negative control.

The effects of mangrove extracts on cellular biomass and the percentage differences with respect to negative controls prepared with 2% MetOH are reported in Figure 3 and Table S3.

Data showed that *B. cylindrica* and *L. racemosa* mangrove extracts did not affect or slightly affected the amount of cellular biomass only in the *E. coli* biofilm. On the contrary, mangrove extracts displayed an opposite effect on *C. albicans* biofilm. Indeed, *B. cylindrica* increased the biofilm biomass at the maximum concentration tested, whereas *L. racemosa* decreased biofilm biomass above 10 mg/L, with an optimal activity at 100 mg/L and 1000 mg/L. In *F. oxysporum*, no effect was shown when the biofilm was grown in the presence of *B. cylindrica*, whereas a decrease was measured in the presence of *L. racemosa* at 100 mg/L and 1000 mg/L.

No differences were shown between the PBS and 2% MetOH negative controls in each of the microorganisms tested.

#### 2.6.2. Mangrove Extracts Increase the Sum and Ratio of Extracellular Polysaccharides and Proteins

**Figure 4:**
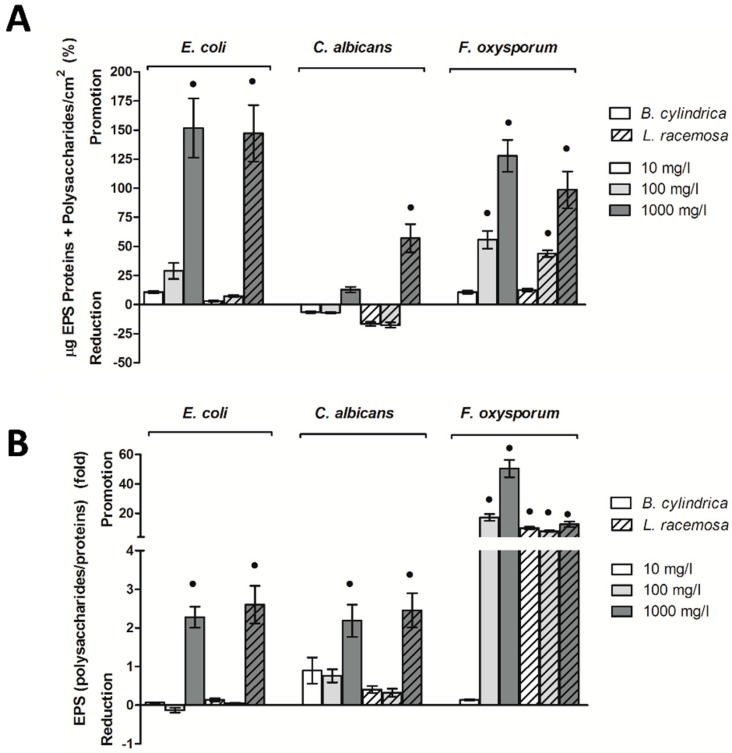
Extracellular polysaccharides and proteins within the biofilm in the presence of *B. cylindrica* and *L. racemosa* extracts. Percentage differences of polysaccharides and proteins sum (**A**) and ratio (**B**) with respect to the negative control plus 2% MetOH are reported. Black dots indicate statistically significant differences with the 2% MetOH negative control.

The abundance of polysaccharides and proteins are reported in Figures S5 and S6 respectively, whereas their sums and ratios are shown in Figure 4 and Table S4.

The results highlighted that the matrix, in terms of proteins plus polysaccharides, increased in the presence of both extracts with respect to the negative controls prepared with 2% MetOH. The highest increase in the polysaccharides and proteins sum was measured in the *E. coli* biofilm when treated with both *B. cylindrica* and *L. racemosa* extracts at the maximum concentrations. In the *C. albicans* biofilm, *L. racemosa* displayed a larger effect than *B. cylindrica.* Indeed, the first increased the ratio at 1000 mg/L, whereas the latter did not affect the ratio value. The sum of extracellular polysaccharides and proteins was also increased in the *F. oxysporum* biofilm when treated with both *B. cylindrica* and *L. racemosa* at 100 mg/L (Figure 4A and Table S4).

The polysaccharides/proteins ratio highlighted that *B. cylindrica* and *L. racemosa* modulated the chemical composition of the biofilm matrix, with a shift toward the increase of exopolysaccharides versus the extracellular proteins. In the *E. coli* and *C. albicans* biofilms, *B. cylindrica* and *L. racemosa* increased the polysaccharides/proteins ratio only at the maximum concentration tested. The effect was more evident in the *F. oxysporum* biofilm. In this biofilm, *B. cylindrica* increased the exopolysaccharides/proteins ratio at 100 mg/L and 1000 mg/L, whereas *L. racemosa* displayed its effect at all the tested concentrations (Figure 4B and Table S4).

No differences in the extracellular polysaccharides and proteins content, sum, and ratio were shown between the PBS and 2% MetOH negative controls, for each of the microorganisms tested.

#### 2.6.3. Mangrove Extracts Increase the Intracellular and Extracellular Amount of Reactive Oxygen Species (ROS)

**Figure 5:**
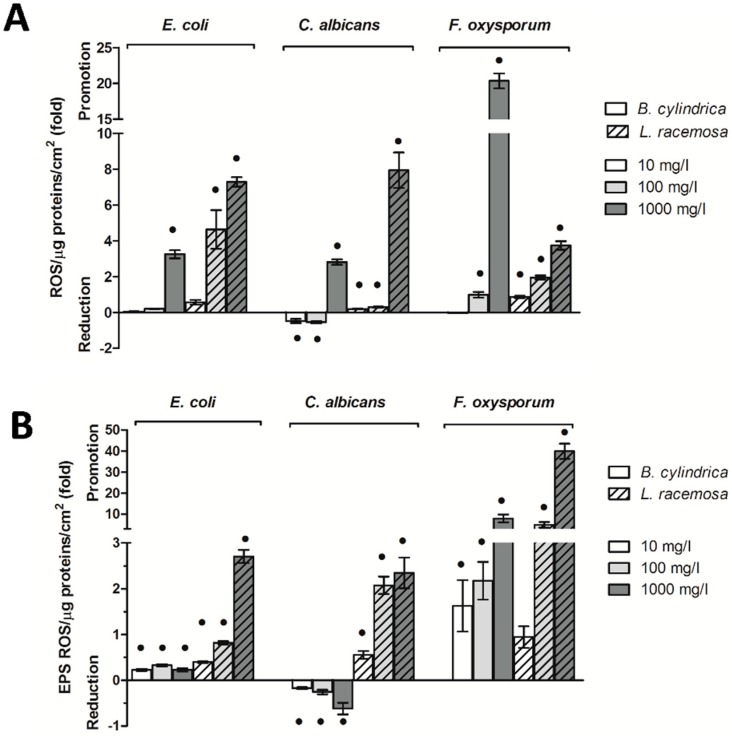
Reactive oxygen species (ROS) within the biofilm in the presence of *B. cylindrica* and *L. racemosa* extracts. In panels (**A**) and (**B**), the percentage differences with respect to the negative control plus 2% MetOH are reported. Black dots indicate statistically significant differences with the 2% MetOH negative control.

The effects of mangrove extracts on the intracellular and extracellular amounts of ROS and the percentage differences in comparison to negative controls prepared with 2% MetOH are shown in Figure 5 and Table S5.

Both mangrove extracts increased the level of intracellular ROS in *E. coli* and *F. oxysporum*, with a major effect of *L. racemosa* in comparison to *B. cylindrica*. The level of intracellular ROS was significantly increased when *E. coli* biofilm was grown in the presence of *B. cylindrica* at 1000 mg/L and *L. racemosa* at 100 mg/L and 1000 mg/L. In *F. oxysporum*, the *B. cylindrica* extract significantly increased the ROS at 1000 mg/L, whereas the *L. racemosa* extract affected the level of ROS at all the concentrations tested. In the *C. albicans* biofilm, both extracts increased the level of ROS at 1000 mg/L. However, at 10 mg/L and 100 mg/L, the level of ROS was significantly abated in the presence of *B. cylindrica* or remained undisturbed with the *L. racemosa* extract.

Similarly to the intracellular ones, extracellular ROS increased in *E. coli* and *F. oxysporum* when biofilms were treated with both mangrove extracts. Indeed, in *E. coli*, the extracellular level of ROS was slightly affected in the presence of *B. cylindrica* at all the concentrations. A high effect was observed in the presence of *L. racemosa* at 100 mg/L and 1000 mg/L. Additionally, both extracts—except for *L. racemosa* at 10 mg/L—significantly increased the extracellular ROS in *F. oxysporum*. *B. cylindrica*, and *L. racemosa* extracts displayed the opposite effect on the *C. albicans* biofilm. Indeed, *B. cylindrica* significantly decreased the extracellular ROS at 1000 mg/L. On the contrary, when the *C. albicans* biofilm was grown in the presence of *L. racemosa*, the extracellular level of ROS increased at 100 mg/L and 1000 mg/L.

For all the microorganisms tested, no differences were shown between the intracellular and extracellular ROS in the PBS and 2% MetOH negative controls.

### 2.7. Mangrove Extract Modulate the Biofilm Detachment

**Figure 6:**
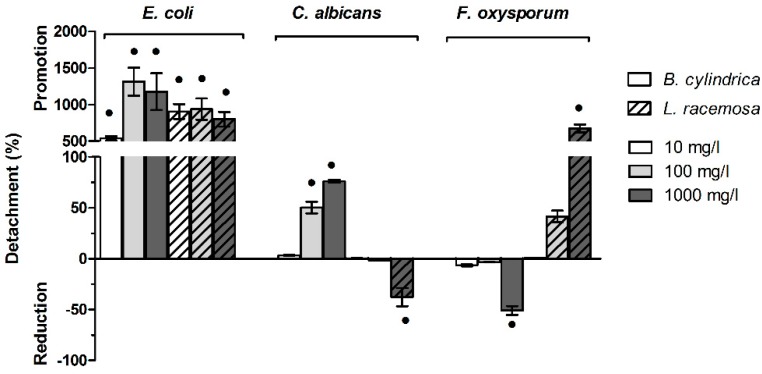
Detachment of biofilm pre-grown without extract and subsequently treated with *B. cylindrica* and *L. racemosa*. Percentage differences with respect to the negative control plus 2% MetOH are reported. Black dots indicate statistically significant differences with the 2% MetOH negative control.

Detachment indexes and the percentage differences with respect to negative controls prepared with 2% MetOH are reported in Figure 6 and Table S6.

When *E. coli* biofilm was pre-grown and subsequently treated with both *B. cylindrica* and *L. racemosa* extracts, the detachment index appeared to increase for all the concentrations tested. Indeed, a maximum increased was observed under the treatment with *B. cylindrica* and *L. racemosa*. As regards *C. albicans* biofilm, the treatment with *B. cylindrica* at 100 mg/L and 1000 mg/L increased the detachment index, whereas the same concentrations of *L. racemosa* did not (100 mg/L) or slightly (1000 mg/L) affected the biofilm detachment. An opposite trend was observed for *F. oxisporum* when treated with mangrove extracts at 1000 mg/L. *B. cylindrica* decreased the detachment index, whereas at the same concentration of *L. racemosa* increased the index.

## 3. Discussion

In this research, we have proved that sub-lethal concentrations of phytochemicals can either inhibit or enhance biofilm formation, and this behavior depends on the microbial taxon and biofilm formation step (Table 2). Thus, plant compounds are used as signaling molecules with regulatory functions [32].

To obtain a general impression of the phytochemical composition of the mangrove material, the total phenol and flavonoid contents as well as the oxygen radical absorbance capacity (ORAC) were analyzed. The crude methanolic leaf extract from *L. racemosa* contained significantly higher amounts of phenols and flavonoids. The ORAC values of both species showed no significant difference, which indicates the presence of antioxidant compounds in *B. cylindrica* that do not belong to the group of phenols. Indeed, *B. cylindrica* is known to contain numerous triterpenoids, which possess antioxidant capacities [33].

Most of the experiments were conducted with the extracts at 10 mg/L, 100 mg/L, and 1000 mg/L concentrations, as these values showed the highest activity on cells/conidia adhesion. In general, the *L. racemosa* extract had a significant bioactivity at lower concentrations than the *B. cylindrica* extract. Disk diffusion assays, planktonic growth experiments, and conidia germination assays were performed with the sole goal of proving that the possible effects on cells/conidia adhesion, biofilm maturation, and detachment were not a consequence of the lethal effect of mangrove extracts, but rather of a specific antibiofilm mechanism that is able to sabotage the propensity for a sessile lifestyle. Moreover, we have shown that at the concentrations tested, mangrove extracts were not a carbon and energy source. Thus, the promotion of microbial biofilm was not due to a supply of nutrients.

The effects of the mangrove extracts were observed on the three steps of the biofilm formation cycle. Adhesion has been reduced in all the models except for *E. coli*, where adhesion was promoted in the presence of the *B. cylindrica* extract. Glasenapp et al. reported an inhibitory activity of tannins extracted from *L. racemosa* against *C. albicans* cell adhesion [21]. The tannin casuarinin was also present in the crude extract used in this study, and was therefore likely important in the observed reduction of biofilm cell adhesion.

In the maturation phase, the mangrove extracts affected cellular biomass quantity in different ways according to the microbial model used. The amount of cellular biomass within the *E. coli* biofilm was not or was slightly affected with the two extracts. At the highest concentrations, *C. albicans* increased its biofilm biomass in the presence of *B. cylindrica*, and decreased biofilm biomass in presence of *L. racemosa*. *F. oxysporum* did not show changes in biofilm biomass in the presence of the *B. cylindrica* extract, but decreased when the *L. racemosa* was present. Among the phenolic compounds and flavonoids identified in the extracts of this study, delphinidin-3-pentoside and delphinidin-3glucoside have been already shown to have activity against mature *Lactobacillus acidophilus* biofilm [34]. Wu et al. [35] found that myricetin, which was also retrieved in mangrove extracts, disintegrated the complex architecture biofilm of *S. aureus* biofilm. Rocha et al. [36] found that the combination of myricetin plus farnesol significantly decreased water-soluble exopolysaccharides in a *C. albicans* biofilm that was 67 hours old.

Many works have claimed that sessile cells produce a biofilm with a highly ordered and complex matrix that gives the three-dimensional structure necessary for biofilm stability [37,38]. The *C. albicans* biofilm matrix mediates adhesive and cohesive interactions, and controls cell detachment from the biofilm [39]. *Fusarium* spp. assembles a self-secreted extracellular matrix (composed also by polysaccharides and proteins) that is fundamental to the biofilm lifestyle, protecting and changing the cell environment [40,41]. For the microorganisms considered in this research, the amount of polysaccharides was always higher than the quantity of proteins (approximately 3:1 in *E. coli*, 4:1 in *C. albicans*, and 20:1 in *F. oxysporum*). At the highest extract concentrations, an increase of the extracellular polymeric substances (EPS) amount—calculated as the sum of exopolysaccharides and extracellular proteins, which was used as a proxy of total EPS [42]—was recorded for all the models with the exception of *C. albicans* with the *B. cylindrica* extract, which showed no change. However, even if the total amount of EPS did not vary, in some cases, the ratio between exopolysaccharides and extracellular proteins changed in favor of an increased amount of polysaccharides. This happened to *C. albicans* and *F. oxysporum* when treated with the *B. cylindrica* and *L. racemosa* extracts at 10 mg/L, respectively. Changes in the structural composition of the biofilm could impact its development even if massive changes in the total amount of EPS are not observed. Indeed, it has been reported that the cohesive energy per unit volume of biofilm strongly correlates with both the polysaccharides concentration and the ratio of polysaccharides to proteins [42]. Polysaccharides were also reportedly involved in *E. coli* adhesion during the transition from planktonic to sessile growth [43].

Among the other extracellular polymeric substances, exopolysaccharides are often engaged in the oxidative stress response [44], as in the case of *Azotobacter vinelandii* [45], *Campylobacter jejuni* [46], or *P. putida* when exposed to oxidative stress [47]. In agreement with the current literature, in this study, the extracts and concentrations involved in the major production of both intracellular and extracellular ROS were always found coupled with a significant shift toward a major production of polysaccharides in the matrix. This effect was evident for all the microbial models, suggesting that exopolysaccharides production could be a common behavior among biofilms to provide tolerance toward environmental stressors. Indeed, in the *E. coli* and *F. oxysporum* biofilms, the trends of intracellular ROS fully overlapped those of extracellular polysaccharides/proteins ratios for all the concentrations of both extracts, whereas in *C. albicans*, this superimposition was evident only at the highest concentration tested. On the contrary, when a reduction of ROS was observed, i.e., in the *C. albicans* biofilm under exposure to *B. cylindrica* at 10 mg/L and 100 mg/L, no shift in the matrix composition was observed. Notably, beside the correlation between ROS and exopolysaccharides that is well known in bacterial biofilms, very few research studies have observed this response in yeast and filamentous fungal biofilms.

Detachment was generally promoted except for *C. albicans* when the *L. racemosa* was present, and for *F. oxysporum* with the *B. cylindrica* extract at 1000 mg/L. In both cases, the detachment observed was the result of a biofilm so thick, it was no longer sufficiently stable.

Over the last few decades, we have learned that microbial biofilms on leaves either positively affect the fitness of the plant and the yield of agricultural crops, or can be the cause of plant diseases [30,48]. Here, we have seen that both the inhibition and stimulation of biofilm steps was possible with the same leave extracts. So far, phytochemicals that promote beneficial biofilm development in the phyllosphere have been less investigated than those that discourage the sessile lifestyle of deleterious microorganisms [49,50,51]. In contrast, it is well known that the plant stimulates the production of beneficial rhizobacterial biofilms on roots through a chemotaxis-mediated response mechanism involving the production of root exudates [52]. Not all microorganisms that come into contact with the phyllosphere are able to colonize the surface and grow. Indeed, a sophisticated machinery allows plants to set up an intimate interaction with microorganisms, providing selective advantage only to specific beneficial microorganisms over the detrimental ones [51]. Among others, attracting mechanisms include the release of a broad range of molecules from leaves, such as phenolic compounds [48,53,54]. For decades, the production of phenolic compounds by leaves has been only associated with plant defense against pathogenic microorganisms [53]. However, notably, these molecules can be either more suitable as a carbon source or produced as signals to recruit only specific taxa and modulate cell genetic and biochemical activities [48,49,55].

## 4. Materials and Methods

### 4.1. Plant Material and Extraction

*L. racemosa* (L.) C.F. Gaertn. and *B. cylindrica* (L.) Blume were grown in the greenhouse of the Institute of Botany, Germany, so that fresh plant material has been always available during this research [21]. The leaves of adult plants were collected in February 2016, directly frozen in liquid nitrogen, and ground to a fine powder. The plant material was extracted with 20 mL of MetOH per 1 g of fresh leaf material. The first extraction step was conducted for 1 h at room temperature (RT) with constant stirring. Afterwards, the extract was filtered (Macherey-Nagel, Düren, Germany) into a round-bottom flask. This step was repeated two times, the first with one-hour duration and the second overnight. The extraction solvent was evaporated in a rotary evaporator (Büchi, Flawil, Switzerland) with 60 °C water bath temperature. The dried extract was re-dissolved in 100% MetOH and filled in reaction tubes, which were subsequently evaporated in a speedvac (Eppendorf, Hamburg, Germany). The reaction tubes were weighed empty and with the dried extract to measure the resulting extract mass. Finally, the extract was dissolved in MetOH to the desired concentration.

### 4.2. Total Flavonoid Content (TFC)

To analyze the total flavonoid content in the extracts from *L. racemosa* and *B. cylindrica*, dilutions of 1 mg/mL were prepared in 80% MetOH. The method used was based on a protocol from Dudonné et al. [56]. In a clear 96-well plate, each well was filled with 150 µL of water. Next, 25 µL of either the extract sample or catechin hydrate standard were added, with five replicates for the extracts and three replicates for the standard. The catechin hydrate calibration standards had the following concentrations: 0 µg/mL, 10 µg/mL, 25 µg/mL, 50 µg/mL, 100 µg/mL, 125 µg/mL, 250 µg/mL, and 400 µg/mL in 80% MetOH. After the addition of 10 µL of NaNO_2_ (3.75%), the plate was incubated at RT for 6 min. Next, 15 µL of AlCl_3_ (10%) was added and incubated for 5 min at RT. In the last step, 50 µL of NaOH (1 M) was added, and the absorption was measured at 510 nm in a microplate reader (Biotek, Winooski, VT, USA). The concentration of the TFC was calculated with the catechin hydrate calibration curve.

### 4.3. Total Phenol Content (TPC)

Dilutions of 1 mg/mL in 80% MetOH of the *L. racemosa* and *B. cylindrica* extracts were used to measure the total phenol content. The protocol was modified based on Dewanto et al. [57]. In each well of a clear 96-well plate, 100 µL water were filled. To the sample and a gallic acid calibration standard, 10 µL was added into the wells, with five replicates for the extracts and three replicates for the standard. The gallic acid calibration standards were used in the following concentrations: 0 µg/mL, 5 µg/mL, 10 µg/mL, 25 µg/mL, 50 µg/mL, 75 µg/mL, 100 µg/mL, 125 µg/mL, and 250 µg/mL in 80% MetOH. First, 10 µL of Folin reagent was added to each well and incubated for 8 min at RT in the dark. In the second step, 100 µL of Na_2_CO_3_ (7%) was added, and the plate was incubated for 100 min in the dark. Finally, the absorption was measured at 765 nm in a microplate reader (Biotek, Winooski, VT, USA). The gallic acid calibration curve was used to calculate the concentration of total phenols.

### 4.4. Oxygen Radical Absorbance Capacity (ORAC)

The ORAC value was measured with diluted mangrove extracts with a concentration of 0.0125 mg/mL in phosphate buffer (75 mM, pH 7.4). This method is based on the protocol described by Huang et al. [58] and Gillespie et al. [59]. In a black 96-well microtiter plate, 120 µL of fluorescein (112 nM) were mixed with 20 µL of mangrove extract or 6-hydroxy-2,5,7,8-tetramethylchroman-2-carboxylic acid (trolox) standard. The extract samples and the trolox standard were analyzed in triplicate. The following concentrations of the trolox calibration standard curve were prepared in phosphate buffer: 6.25 µM, 12.5 µM, 25 µM, and 50 µM. Then, the plate was incubated for 15 min at 37 °C, and after the incubation time, the fluorescence was measured at 485/520 nm as time point zero. In the next step, 80 µL of 2,2′-azobis(2-amidino-propane) dihydrochloride (62 mM) was added to each well, and the fluorescent signal was measured over 80 min at 485/520 nm. The ORAC value was calculated using the trolox calibration curve with the difference between the signal at time point zero and 80 min.

### 4.5. LC-MS Analysis

For LC-MS analysis, an HPLC (Shimadzu, Darmstadt, Germany) coupled to a triple TOF mass spectrometer (AB Sciex, Canby, USA) was used. Water (A) and methanol (B) supplemented with 2 mM of ammonium acetate and 0.01% acetic acid were used as solvents. The flow rate was 0.8 mL/min, and a gradient was applied with 10% to 90% B over 35 min, two min of 90% B, and switching to 10% B in one min, followed by two min of 10% B. The column oven temperature was 30 °C, and 10 µL of the sample was injected into a Knauer Vertex Plus column (250 × 4 mm, 5 µm particle size, packing material ProntoSIL 120-5 C18-H) with precolumn (Knauer, Berlin, Germany). The ion source was set to negative electrospray ionization mode (ESI–) and mass spectra were recorded between 100–1000 Da. MS/MS spectra were measured at a collision energy of −30 eV between 50–800 Da. Precursor masses and MS/MS peaks were compared to database entries in MassBank [60] and ReSpect [61] for identification.

### 4.6. Microbial Strains and Growth Media

*E. coli* ATCC 25404, *C. albicans* ATCC MYA-2876, and *F. oxysporum* D221 (from the collection of the Department of Food, Environmental, and Nutritional Sciences, Università degli Studi di Milano collection) were used as model systems for the bacterial, yeast, and filamentous fungal biofilms, respectively. *E. coli* was chosen, being a K-12 cosmopolitan strain [62]; *C. albicans* is a widespread polymorphic yeast fungus responsible of important human infections [63], whereas *F. oxysporum* is in the top 10 fungal plant pathogen list, which is accountable for serious crop losses around the world [64].

The strains (conidia for *F. oxysporum*) were stored at −80 °C in suspensions containing 20% glycerol and 2% peptone.

*E. coli* was grown in tryptic soy broth (TSB, Sigma Aldrich, USA) at 30 °C for 24 h, *C. albicans* was grown in yeast nitrogen base broth supplemented with 50 mM of glucose (YNBg, Sigma Aldrich, USA) at 30 °C for 24 h, and *F. oxysporum* was grown on potato dextrose agar (PDA, Sigma Aldrich, USA) at 21 °C for 7 days. After being routinely grown, *E. coli* and *C. albicans* cells were washed three times in PBS (Sigma Aldrich, St. Louis, MO, USA) and used in the subsequent experiments. *F. oxysporum* conidia were collected by suspending them in PBS and filtering using a double-layered sterile gauze. A light microscope (Leica DM4000 M, Leica Microsystems, Wetzlar, Germany) and a Thoma counting chamber have been used to quantify *E. coli* cells, *C. albicans* cells, and *F. oxysporum* conidia concentration.

The pH of each medium added with each extract was evaluated employing a Jenway 3510 pH meter (pH 7.5).

### 4.7. Mangrove Extract Toxicity

#### 4.7.1. Biocidal Activity

The disk diffusion assay has been used to evaluate the biocidal activity of mangrove extracts [65]. First, 200 µL of a suspension of 10^6^ *E. coli* cells, *C. albicans* cells, or *F. oxysporum* conidia were spread uniformly on Petri plates (80 mm diameter) filled with tryptic soy agar (TSA, Sigma Aldrich, USA) for *E. coli*, YNBg agar medium for *C. albicans* and PDA for *F. oxysporum*. Filter-paper discs (6-mm diameter, Oxoid, United Kingdom) were soaked with 0.001 mg/L, 0.01 mg/L, 0.1 mg/L, 1 mg/L, 10 mg/L, 100 mg/L, and 1000 mg/L of both extracts with 2% MetOH. Then, the discs were put in the center of the Petri dishes. Negative controls were obtained by loading filter paper discs with 20 µL of PBS or 2% MetOH instead of each extract, whereas positive controls were performed by loading filter paper discs with 20 µL of chloramphenicol (30 µg/mL in water, Sigma Aldrich, USA), sodium hypochlorite (5% in water, Sigma Aldrich, USA), or Procloraz (0.01% in water, Sportak, BASF, Germany) for *E. coli*, *C. albicans*, and *F. oxysporum*, respectively. *E. coli* and *C. albicans* were incubated at 30 °C for 24 h, whereas *F. oxysporum* was incubated 21 °C for 7 days. In case the extracts inhibited microbial growth, a halo was present around the disks [65].

#### 4.7.2. Modulation of Planktonic Growth

*E. coli* and *C. albicans* planktonic growth assays were carried out according to Cattò et al. [66]. Briefly, 10^6^ cells/mL of *E. coli* and *C. albicans* were grown respectively in TSB or YNBg medium supplemented with 0.001 mg/L, 0.01 mg/L, 0.1 mg/L, 1 mg/L, 10 mg/L, 100 mg/L, and 1000 mg/L of both extracts with 2% MetOH in 96-well microtiter plates at 30 °C. Negative controls were prepared by growing microbial strains in TSB or YNBg medium supplemented with PBS or 2% MetOH. Microbial growths were obtained by measuring the absorbance at 600 nm (A_600_) every 15 min for over 24 h using the Infinite 200 PRO Microplate Reader (Tecan, Männedorf, Switzerland). Absorbance-based growth kinetics were obtained considering the A_600_ of suspensions minus the A_600_ of the non-inoculated medium as a function of time.

*F. oxysporum* planktonic growth was assessed by measuring the colony radial growth. Briefly, 6-mm diameter discs were cut from the peripheral region of a 7-day-old *F. oxysporum* culture and transferred to other plates prepared with PDA added with 0.001 mg/L, 0.01 mg/L, 0.1 mg/L, 1 mg/L, 10 mg/L, 100 mg/L, and 1000 mg/L of both extracts supplemented with 2% MetOH. Negative controls were prepared by growing fungal strain in PDA supplemented with PBS or 2% MetOH. Growth was recorded by measuring the radius of colonies in four directions every 24 h for 9 days. Growth kinetic curves were constructed by plotting the average values of the radius against the time.

The polynomial Gompertz model adapted for microbial growth by Zwietering [67] was used to fit the growth curves, and the λ, the μ_m_, and the YM were determined employing the GraphPad Prism software (version 5.0, San Diego, CA, USA). Three biological replicates were performed for each treatment, and four technical replicates were carried out for each experiment.

#### 4.7.3. Effect on Conidia Germination

The ability of mangrove extracts to modulate the germination of *F. oxysporum* conidia was tested by plating 30 µL of 10^6^/mL conidia on PDA with the addition of 0.001 mg/L, 0.01 mg/L, 0.1 mg/L, 1 mg/L, 10 mg/L, 100 mg/L, and 1000 mg/L of each extract and 2% MetOH. Negative controls were prepared by plating conidia in PDA with the addition of only PBS and 2% MetOH. After 21 h of incubation at 21 °C, the germinated conidia percentage was determined by direct microscope counting (10 random view fields for each replicate).

### 4.8. Mangrove Extracts as Carbon and Energy Source

The ability of *E. coli* and *C. albicans* to grow with mangrove extract as the sole energy and carbon source was evaluated according to Cattò et al. [68]. Briefly, 10^6^ cells/mL were grown in a mineral medium (KH_2_PO_4_ 30 g/L, Na_2_HPO_4_ 70 g/L, NH_4_Cl 10 g/L) prepared with the addition of 1000 mg/L of each extract and 2% MetOH. The incubation was carried out at 30 °C for 48 h. Then, the A_600_ was estimated by a UV/VIS 7315 spectrophotometer (Jenway, Essex, UK) and compared with controls. The positive controls were prepared by growing microbial strains in the mineral medium supplemented with 30 g/L of sucrose, whereas the negative controls were prepared by growing them in the mineral medium supplemented with PBS or 2% MetOH.

The ability of *F. oxysporum* to grow with the mangrove extracts as a unique carbon source was investigated by inoculating 200 µL of 10^6^/mL conidia on Petri dishes filled with a mineral medium agar (3 g/L of NaNO_3_, 1 g/L of K_2_HPO_4_, 0.5 g/L of MgSO_4_x7H_2_O, 0.5 g/L of KCl, and 0.01 g/L of FeSO_4_·7H_2_O) with the addition of 1000 mg/L of each extract and 2% MetOH as the unique energy and carbon source, respectively. Negative controls were also prepared with the addition of only PBS or 2% MetOH, whereas positive controls were performed with the addition of 30 g/L sucrose. Fungi were incubated at 21 °C for 7 days. The fungal growth was investigated by comparison with controls.

Mangrove extracts were the only possible source of energy and carbon available for the microbial strains. Therefore, microbial growth was possible only in case microorganisms were able to use the added extract as energy and carbon source.

### 4.9. Mangrove Effect on Cells/Conidia Adhesion

*E. coli* and *C. albicans* cells or *F. oxysporum* conidia adhesion was evaluated in hydrophobic black-sided plates. Briefly, 200 μL of PBS containing 10^6^ cells or conidia with the addition of 0.001 mg/L, 0.01 mg/L, 0.1 mg/L, 1 mg/L, 10 mg/L, 100 mg/L, and 1000 mg/L of each extract and 2% MetOH were placed in microtiter plate wells and incubated at 30 °C (*E. coli* and *C. albicans*) or 21 °C (*F. oxysporum*). Experiments were also carried out with PBS and 2% MetOH as negative controls. The incubation lasted 24 h. Then, the wells were washed twice with 200 μL of PBS, and adhered cells or conidia were stained with either 4′,6-diamidino-2-phenylindole (*E. coli*) (Sigma Aldrich, USA) [68] or Fluorescent Brightener 28 (*C. albicans* and *F. oxysporum*) (Sigma Aldrich, USA) [69] for 20 min in the dark at room temperature. The Infinite 200 PRO Microplate Reader (Tecan, Männedorf, Switzerland) was employed to measure the fluorescence intensity under a 335-nm excitation wavelength and a 433-nm emission wavelength. The number of adhered cells or conidia was calculated using a standard curve of fluorescence intensity. After data normalization to the area, the means were reported. Three biological replicates were performed for each treatment, and at least eight technical replicates were performed for each experiment. Only the best anti-adhesion sub-lethal concentrations were used in the subsequent experiments to further study the mangrove effect on biofilm maturation.

### 4.10. Mangrove Effect on Biofilm Maturation

#### 4.10.1. Biofilm Culture

Two hundred µL of 10^6^ *E. coli* cells, *C. albicans* cells, or *F. oxysporum* conidia were inoculated in Petri dishes (35-mm diameter) containing 2 mL of 10% diluted TBS for *E. coli*, 10% diluted YNBg for *C. albicans*, and 10% diluted potato dextrose broth (PDB) for *F. oxysporum* with the addition of 10 mg/L, 100 mg/L, and 1000 mg/L of each extract and 2% MetOH. Negative controls were performed by growing biofilms with only PBS or 2% MetOH. Petri dishes containing *E. coli* and *C. albicans* were incubated at 30 °C for 48 h, whereas those containing *F. oxysporum* were placed at 21 °C for 7 days, allowing mature biofilm development. The media were replaced every 48 h to avoid any change in the experimental conditions, and consequently of the biofilm growth rate due to nutrient deficiency and the build-up of metabolic products [70].

#### 4.10.2. Biomass and EPS Extraction

After incubation, the media were displaced from the Petri dishes, and the biofilm was resuspended in 1 mL of 2% ethylenediaminetetraacetic acid (EDTA) and transferred into new tubes. To avoid aggregates, biofilm suspensions were homogenized by a 30-s cycle at 14,500 rpm (T 10 basic Ultra-Turrax) and sonicated for 15 s (15% amplitude, in water-bath; Branson 3510, Branson Ultrasonic Corporation, Dunburry, CT) and 30 s of vortex mixing. EPS was extracted from biofilm suspensions after an overnight incubation at 4 °C with gentle shaking (300 rpm). Then, biofilm suspensions were centrifuged (11,000× *g*) for 30 min at 4 °C. The EPS-containing supernatants were filtered using a 0.2-µm filter, while the pellets were used in further experiments (see Section 4.10.3 and Section 4.10.5).

#### 4.10.3. Biomass Abundance

Cellular biomass was indirectly estimated by quantifying cellular proteins under the assumption that their content is similar between cells [71].

EDTA traces were removed, washing the pellets with distilled water twice. Then, cells were resuspended in 1 mL of PBS. Samples were transferred in screw-cap 2-mL vials containing approximately 100 μL of glass beads (diameter <106 μm and between 425–600 μm, Sigma Aldrich, USA). Cells were subjected to a mechanical disruption using a Precellys bead beater (Bertin Instrument, Montigny-le-Bretonneux, France) performing six cycles of 30 s at 6500 rpm, with a 30-s period of cooling between cycles. Then, each sample was centrifuged (11,000× *g*) for 30 min at 4 °C. The supernatants were recovered, and the proteins that were released from the broken cells were quantified by the Bradford assay [72] using bovine serum albumin as standard. After data normalization to the area, the means were reported.

#### 4.10.4. Extracellular Polymeric Substances (EPS) Composition

The phenol–sulfuric acid assay was applied for polysaccharides evaluation using glucose as the standard [73], whereas the Bradford method [72] was applied to analyze the protein concentrations in the filtered soluble EPS. Absorbance was determined using a UV/VIS 7315 spectrophotometer (Jenway, Essex, UK). After data normalization to the area, the polysaccharides and proteins abundance and their sums and ratios were reported [42].

#### 4.10.5. Level of Intracellular and Extracellular Oxidative Stress

The 2′,7′-dichlorodihydrofluorescein diacetate (H_2_DCFDA) assay was used to determine the oxidative stress level within the cellular biomass and the EPS component [74]. Briefly, 300 µL of each sample was incubated at 30 °C with 5 mM of H_2_DCFDA (final concentration of 10 µM). After 30 min, fluorescence was assessed using the Infinite 200 PRO Microplate Reader (Tecan, Männedorf, Switzerland) with excitation at 488 nm and emission at 520 nm. Obtained data were normalized to the amount of proteins, and the means are reported.

### 4.11. Mangrove Effect on Biofilm Dispersion

Mature biofilms were grown as reported in Section 4.10.1 in the absence of mangrove extract. After being grown, the media were removed, and the biofilms were treated with 1 mL of PBS containing 10 mg/L, 100 mg/L, and 1000 mg/L of each extract and 2% MetOH. Biofilms were also treated with only PBS or 2% MetOH for negative controls. After 3 h at room temperature, biofilms were dispersed in the bulk, and those that remained on the surface were recovered, and the biomass was indirectly estimated by protein quantification, as reported in Section 4.10.3. The biofilm tendency to surface detachment was calculated as (proteins from biofilm dispersed in the bulk × 100)/(proteins from biofilm dispersed in the bulk + proteins from biofilm remained on the surface) [75], and means were reported.

### 4.12. Statistical Analysis

If not specified otherwise, three biological replicates for each treatment and three technical replicates were carried out for each experiment, and the data were reported as mean ± standard deviation.

The percentage variation of cells/conidia adhesion, cellular biomass abundance, extracellular proteins and polysaccharides, and detachment index in comparison to the negative control prepared with 2% MetOH was calculated as (treated sample data − negative control data) × 100/negative control data. With respect to the negative control, mangrove extract concentrations that were able to affect the above biofilm parameters by less than 20% were considered without activity, and with low, moderate, and excellent activity in the ranges of 20% to 30%, 30% to 50%, and >50%, respectively [68].

First, we verified that the data satisfied the assumptions of (i) independence, (ii) normal distribution, and iii) homogeneity of variance. Then, using MATLAB (Version 7.0, The MathWorks Inc., Natick, MA, USA), the analysis of variance (ANOVA) was applied to statistically evaluate significant differences among the samples. Tukey’s honestly significant different test (HSD) was used for pairwise comparison to determine the significance of the data (*p* < 0.05).

## 5. Conclusions

In this work, we proved that each new compound or extract needs to be investigated, taking into account the multiple steps of biofilm formation, in order to assess its real behavior in the environment. Consequently, this study adds another layer of complexity to the mode of action of plant extracts, supporting that it is not sufficient information that some of the literature on a specific extract shows its antibiofilm activity or biofilm promotion. Instead, the phytochemical effects have to be proved on the target microorganism in all three phases of biofilm development.

The behavior of different extracts on the microbial taxon could be positively exploited in those fields where it is necessary at the same time to reduce some microbial strains and improve the development of others.

## Figures and Tables

**Table 1:** Individual compounds identified by comparison of MS/MS spectra with database entries in *B. cylindrica* leaf extract and *L. racemosa* leaf extract.

***B. Cylindrica* Leaf Extract**						
**No**	**RT**	**Mass**	**MS/MS**	**Name**	**Accession**	**Source**
1	2.5	341.11	179.0, 161.0, 119.0, 89.0, 71.0	Galactinol	PT211910	ReSpect
2	7.8	353.08	191.05, 179.03, 135.04	Caffeic acid mod.	-	-
3	9.1	372.13	132.04, 59.01	n. i.	-	-
4	11.0	427.18	367.16, 221.10	Hirsutine	TY000056	MassBank
5	11.9	456.15	310.09, 148.04, 132.04, 121.03	n. i.	-	-
6	14.2	535.16	475.14, 323.09, 221.06, 179.05, 151.04	n. i.	-	-
7	15.8	445.2	385.18, 205.12, 161.04, 153.09	1-O-b-D-glucopyranosyl sinapate mod.	PS118108	ReSpect
8	17.1	475.18	415.16, 269.10, 161.04	n. i.	-	-
9	19.2	447.09	429.08, 357.06, 327.05, 285.04	Homoorientin	PT204250	ReSpect
10	19.8	577.16	413.09, 311.05, 293.04	Vitexin-2″-O-rhamnoside	PT208750	ReSpect
11	21.0	577.15	413.09, 311.05, 293.04	Vitexin-2″-O-rhamnoside Isomer	PT208750	ReSpect
12	24.3	461.11	341.06, 299.05, 283.02	n. i.	TY000253	MassBank
13	31.4	293.17	236.10, 221.15, 205.12	n. i.	-	-
14	32.0	339.22	307.19, 289.18, 245.19	n. i.	-	-
***L. racemosa* Leaf Extract**						
**No**	**RT**	**Mass**	**MS/MS**	**Name**	**Accession**	**Source**
1	2.7	181.07	163.06, 119.03, 101.02, 89.02, 71.01	Mannitol	PT211960	Respect
2	2.9	341.10	179.05, 161.04, 119.03, 89.02	Galactinol	PT211910	Respect
3	5.3	331.06	271.04, 211.02, 169.01, 151.00	n. i.		
4	8.4	305.06	261.07, 219.06, 165.01, 125.02	(–)-Epigallocatechin	ML000151	MassBank
5	10.7	467.03	458.03, 343.01, 301.00, 275.02, 249.04, 169.01, 125.02	Casuarinin	-	[21]
6	11.7	305.07	225.11, 96.96	n. i.	-	-
7	13.3	183.03	168.00, 124.01, 106.00, 78.01	(galloyl) Methyl gallate	PM012531, PM012533	Respect
8	14.2	457.08	305.06, 169.01, 125.02	Epigallocatechin 3,5-digallate	43930	Metlin
9	15.0	319.04	301.03, 257.04, 215.03, 193.01, 175.00, 125.02	Delphinidin	Standard	
10	16.1	303.09	96.96, 79.95	n. i.	-	-
11	21.1	463.09	316.02, 287.02, 271.02	Myricitrin	PR040144	MassBank
12	22.2	601.09	449.07, 316.02, 179.00	Myricetin-3-xyloside glycoside	PS093009	Respect
13	22.9	615.10	463.08, 317.03, 179.00	Myricitrin glycoside	PR040144	MassBank
14	24.8	423.00	343.05, 328.02, 313.00, 297.97	Robinetin trimethyl ether mod.	BML01849	MassBank
15	31.4	293.17	236.10, 221.15, 205.12, 192.11	n. i.	-	-

No = number of peak in Figure S1, RT = retention time, Mass = mass of precursor ion, MS/MS= fragment spectra obtained at −30 eV, Accession = accession number in database, Source = database used, n. i. = not identified, mod. = modified.

**Table 2:** Summary of the effects of *B. cylindrica* and *L. racemosa* on the three steps of *E. coli*, *C. albicans*, and *F. oxysporum* biofilm formation. Increase (+), decrease (–), and no differences (=) in comparison to the negative control prepared with 2% MetOH with the addition of each microbial strain. EPS: extracellular polymeric substances.

	*E. coli*		*C. albicans*		*F. oxysporum*	
	*B. cyl.*	*L. rac.*	*B. cyl.*	*L. rac.*	*B. cyl.*	*L. rac.*
**ADHESION**	+	−	−	−	−	−
**MATURATION**						
**Cellular biomass**	−	−	+	−	=	−
**EPS (polysaccharides + proteins)**	+	+	=	+	+	+
**EPS (polysaccharides/proteins)**	+	+	+	+	+	+
**Intracellular ROS**	+	+	+	+	+	+
**Extracellular ROS**	+	+	−	+	+	+
**DETACHMENT**	+	+	+	−	−	+

## Supplementary Materials

Supplementary materials can be found at https://www.mdpi.com/1422-0067/20/14/3549/s1.
