# Supplementary material for: Promoting Beneficial and Inhibiting Undesirable Biofilm Formation with Mangrove Extracts
Source: Int J Mol Sci. 2019 Jul 19;20(14):3549. doi: 10.3390/ijms20143549 (PMC6678755; doi:10.3390/ijms20143549)
Supplement: Supplementary file 1 [file ijms-20-03549-s001.pdf]

Supplementary Materials

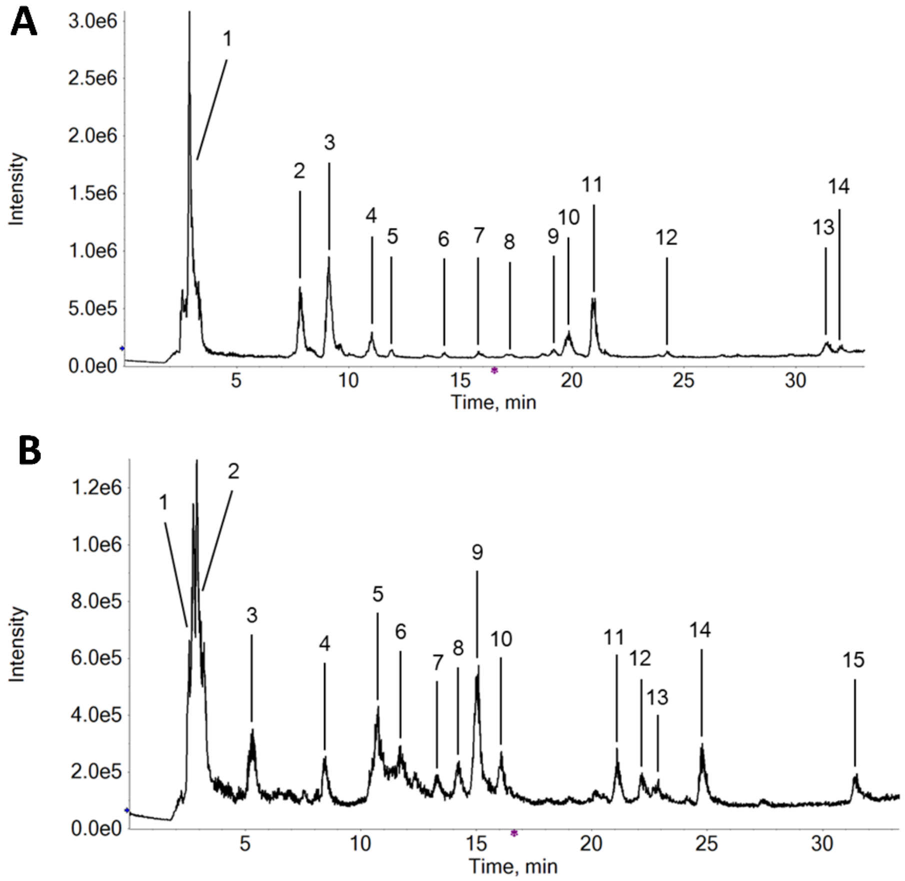

Figure S1. LC-MS chromatograms from *B. cylindrica* extract (panel A) and *L. racemosa* extract (panel B) from minute 0 to 33. The relative intensity of masses between 100 and 800 Da is shown. Numbers indicate substances identified by database entries and are reported in Table 1.

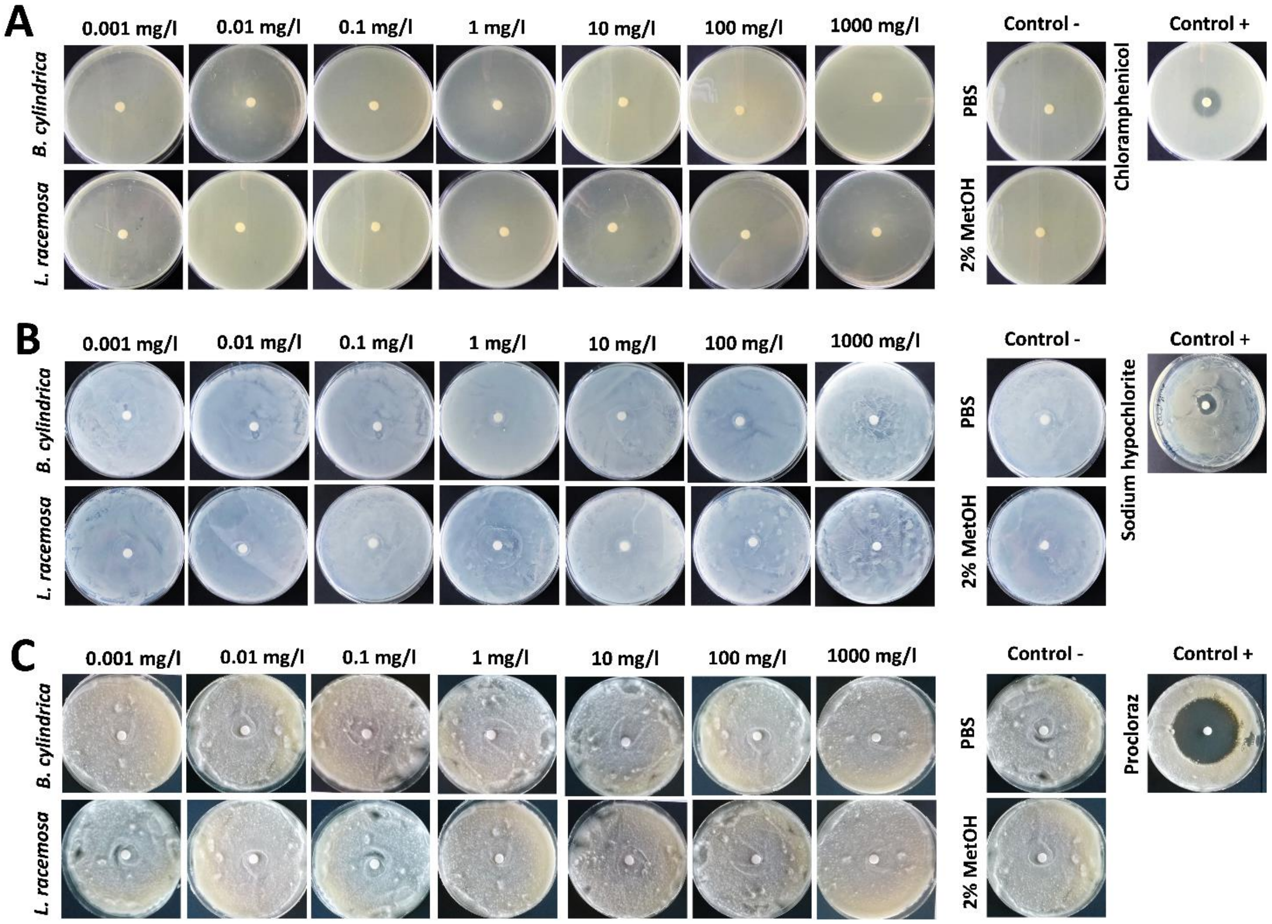

Figure S2. Representative pictures of *E. coli* (A), *C. albicans* (B) and *F. oxysporum* (C) grown in Petri plates with filter-paper discs at the center imbibed with *B. cylindrica* or *L. racemosa* extracts at different concentrations. Filter paper discs were also prepared with PBS or 2% MetOH as negative controls and the positive controls.

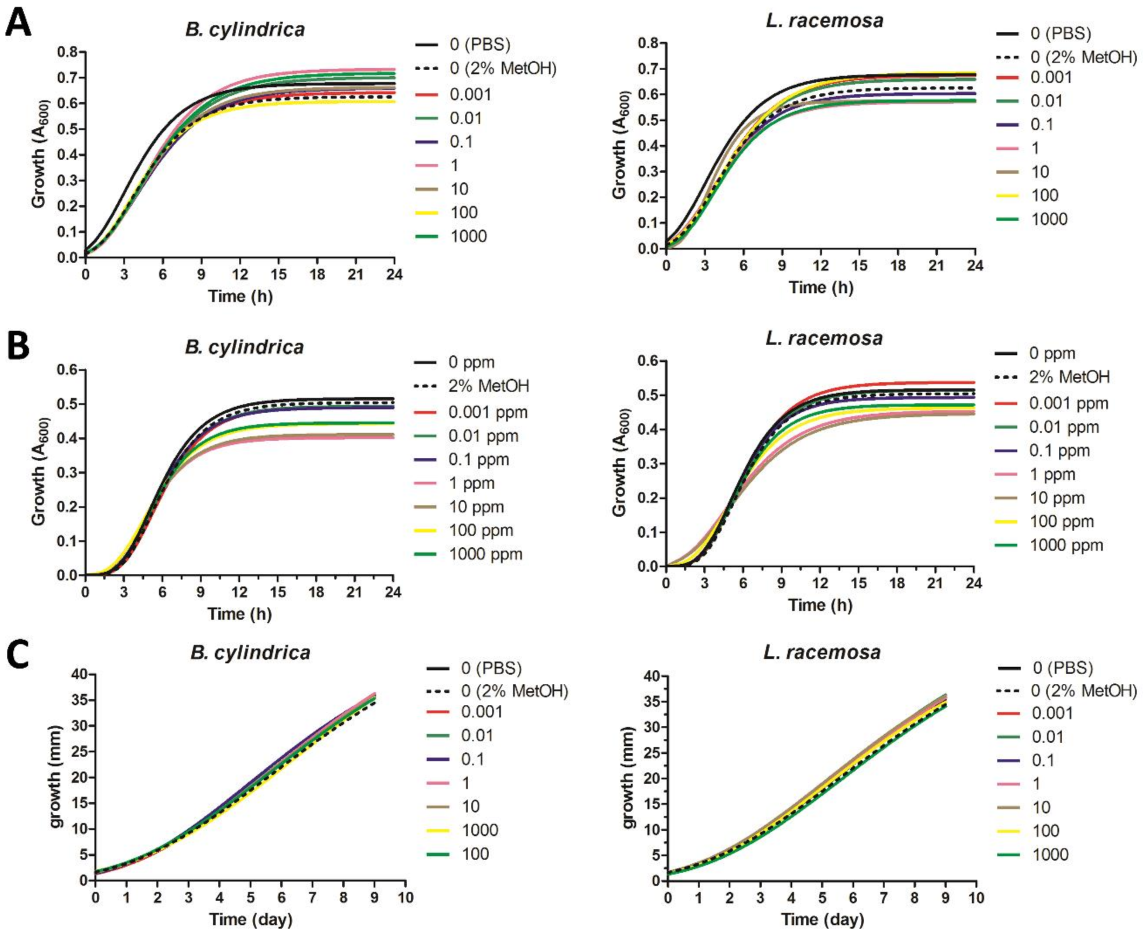

Figure S3. *E. coli* (A), *C. albicans* (B) and *F. oxysporum* (C) planktonic growth curves without and with *B. cylindrica* or *L. racemosa* extracts at different concentrations.

A

	E. coli	*C. albicans*
**1000 mg/l *B. cylindrica***	0.028±0.04 a	0,033±0.05 a
**1000 mg/l *L. racemosa***	0.029±0.03 a	0,034±0.06 a
**Negative control (PBS)**	0.031±0.003 a	0,035±0.08 a
**Negative control (2% MetOH)**	0.032±0.002 a	0.033±0.07 a
**Positive control (30 g/l saccharose)**	0.153±0.04 b	0.358±0.10 b

B

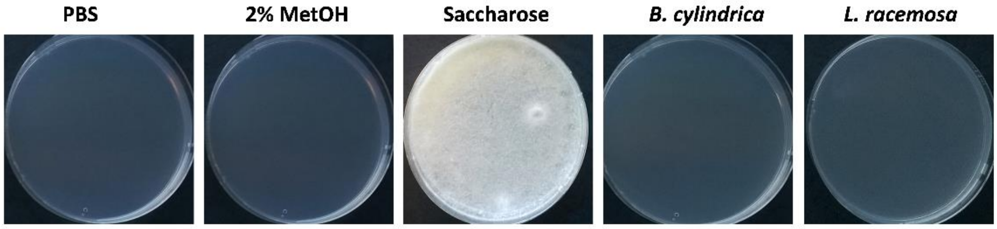

Figure S4. *E. coli* (A), *C. albicans* (A) and *F. oxysporum* (B) planktonic growth in the presence of *B. cylindrica* and *L. racemosa* at 1000 mg/l as the sole carbon and energy source. In panel A data represent the mean ± standard deviation of three independent measurements of A_{600} and different superscript letters indicate statistically significant differences (Tukey's HSD, p ≤0.001) among the samples. In panel C representative pictures of *F. oxysprum* grown in mineral medium agar in the presence of *B. cylindrica* and *L. racemosa* as the sole carbon and energy source are reported. The positive controls were the mineral medium supplemented with 30 g/l sucrose whereas the negative controls were prepared with the mineral medium supplemented with PBS or 2% MetOH.

A

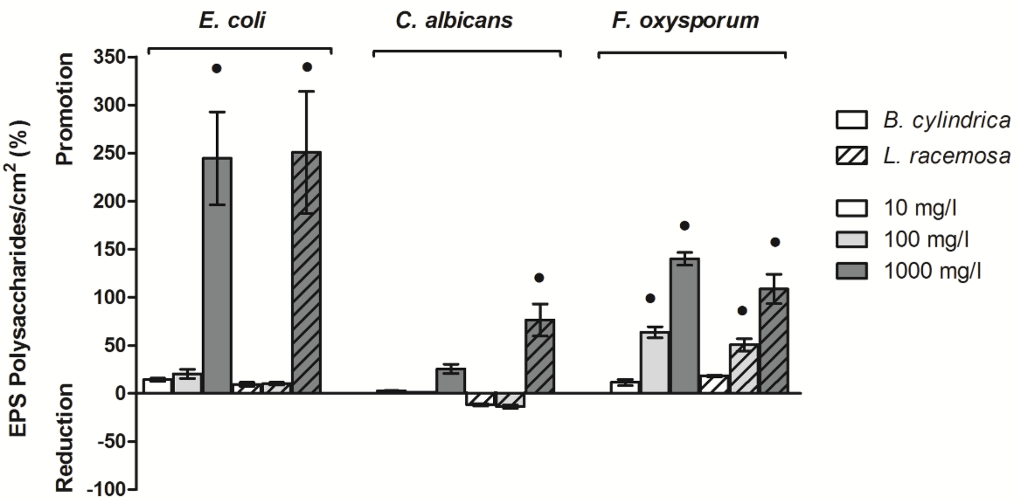

B

	Polysaccharides (µg /cm²)					
	E. coli		***C. albicans***		***F. oxysporum***	
**Conc. (mg/l)**	***B. cylindrica***	***L. racemosa***	***B. cylindrica***	***L. racemosa***	***B. cylindrica***	***L. racemosa***
**0 (PBS)**	1.01±0.06 a	1.01±0.06 a	2.42±0.54 a	2.24±0.54 a	8.68±1.25 a	8.68±1.25 a
**0 (2% MetOH)**	0.97±0.19 a	0.97±0.19 a	2.41±0.25 a	2.41±0.25 a	8.27±1.35 a	8.27±1.35 a
**10**	1.11±0.12 a	1.06±0.23 a	2.47±0.28 a*	2.12±0.23 a*	9.21±1.13 a	9.75±0.98 a
**100**	1.17±0.28 a	1.07±0.14 a	2.44±0.22 a*	2.08±0.24 a*	13.53±1.81 b (+63.6±5.8)	12.45±0.80 b (+50.6±6.3)
**1000**	3.34±0.66 b (+3.4±0.5 fold)	3.40±0.86 b (+3.5±0.6 fold)	3.03±0.59 a*	4.25±0.93 b* (+76.4±16.7)	19.84±2.11 c (+2.4±0.1 fold)	17.24±2.73 c (+2.1±0.1 fold)

Figure S5. Extracellular polysaccharides within the biofilm in the presence of *B. cylindrica* and *L. racemosa* extracts. In panel A percentage differences with respect to the negative control prepared with the addition of 2% MetOH is reported. Dots indicate statistically significant differences with the 2% MetOH negative control. Panel B shows µg of EPS polysaccharides/cm² in the presence of each extract. Different letters indicate statistically significant differences (Tukey's HSD, $p \leq 0.01$) between the different concentrations, whereas an asterisk indicates a significant difference in the EPS polysaccharides between *B. cylindrica* and *L. racemosa* at the same concentration. In the brackets, percentage increase in comparison to 2% MetOH control sample is reported when significant. Data represent the mean ± standard deviation of at least three independent measurements.

Data showed that *B. cylindrica* and *L. racemosa* mangrove extracts significantly increased the quantity of polysaccharides within the biofilm matrix in all microbial strains. *E. coli* showed the maximum increase at the highest concentration tested, up to 3.5±0.6 fold in comparison to the 2% MetOH negative control. Exopolysaccharides did not change when *C. albicans* was grown with *B. cylindrica* extract, whereas an increase of 76.4±16.7% was found in the presence of *L. racemosa* at 1000 mg/l. In *F. oxysporum*, mangrove extract affected the amount of EPS polysaccharides with an increase up to 63.6±5.8% at 100 mg/l, and up to 2.4±0.1 fold at 1000 mg/l, in comparison to the 2% MetOH negative control.

At same concentrations, *L. racemosa* and *B. cylindrica* activity was comparable in *E. coli* and *F. oxysporum*, whereas *L. racemosa* was better than *B. cylindrica* in *C. albicans* biofilm.

No differences were shown between the PBS and 2% MetOH negative controls, in each of the microorganisms tested.

A

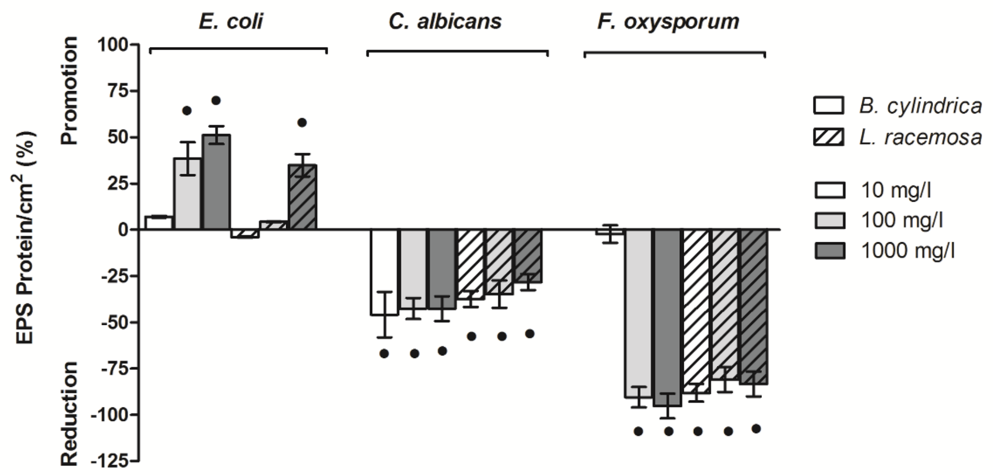

B

	Proteins (μg /cm²)					
	E. coli		*C. albicans*		*F. oxysporum*	
**Conc. (mg/l)**	***B. cylindrica***	***L. racemosa***	***B. cylindrica***	***L. racemosa***	***B. cylindrica***	***L. racemosa***
**0 (PBS)**	0.89±0.15 a	0.89±0.15 a	0.54±0.05 a	0.54±0.05 a	0.43±0.03 a	0.43±0.03 a
**0 (2% MetOH)**	0.89±0.11 a	0.89±0.11 a	0.55±0.06 a	0.55±0.06 a	0.45±0.12 a	0.45±0.12 a
**10**	0.95±0.07 ab	0.86±0.09 a	0.30±0.08 b (-45.9±12.3)	0.34±0.04 b (-37.4±4.3)	0.44±0.02 a*	0.05±0.02 b* (-88.1±4.8)
**100**	1.23±0.29 bc* (+38.4±8.9)	0.93±0.05 a	0.31±0.04 b (-42.6±5.7)	0.36±0.08 b (-34.8±7.4)	0.04±0.02 b* (-90.5±5.5)	0.09±0.03 b* (-80.9±6.7)
**1000**	1.35±0.13 c (+51.1±4.8)	1.20±0.21 b (+32.8±6.1)	0.31±0.05 b* (-42.6±6.7)	0.39±0.06 b* (-28.3±4.3)	0.02±0.03 b* (-95.3±6.7)	0.08±0.03 b* (-83.3±6.7)

Figure S6. Extracellular proteins within the biofilm in the presence of *B. cylindrica* and *L. racemosa* extracts. In panel A percentage differences with respect to the negative control prepared with the addition of 2% MetOH is reported. Dots indicate statistically significant differences with the 2% MetOH negative control. Panel B shows μg of EPS proteins/cm² in the presence of each extract. Different letters indicate statistically significant differences (Tukey's HSD, $p \leq 0.01$) between the different concentrations, whereas an asterisk indicates a significant difference in the EPS proteins between *B. cylindrica* and *L. racemosa* at the same concentration. In the brackets, percentage reduction/increase in comparison to 2% MetOH control sample is reported when significant. Data represent the mean ± standard deviation of at least three independent measurements.

B. cylindrica and *L. racemosa* mangrove extracts showed a different effect depending on microbial model. At 1000 mg/l both extracts increased the *E. coli* extracellular proteins, with moderate activity when the biofilm was grown with *B. cylindrica* and up to 51.1±4.8% with *L. racemosa*. At 100 mg/l *B. cylindrica* did not affect *E. coli* extracellular proteins, whereas a low effect was found when the biofilm was treated with *L. racemosa* at the same concentration. On the contrary, *B. cylindrica* and *L. racemosa* decreased the amount of proteins in the biofilm matrix of *C. albicans* and *F. oxysporum*. The effect was highly significant in *F. oxysporum*, which shown an excellent decrease in the amount of extracellular proteins when treated with B. *cylindrica* at 100 and 1000 mg/l (up to -95.3±6.7%) and *L. racemosa* at 10, 100 and 1000 mg/l (up to -88.1±4.8%). In *C. albicans*, both extracts displayed a moderate effect at all the concentration tested, up to -45.9±12.3% when the biofilm was grown with *B. cylindrica*, and up to - 34.8±7.4% when the biofilm was grown with *L. racemosa*.

In all strains, the experiments showed a major effect of *B. cylindrica* in comparison to *L. racemosa* in increasing or decreasing the quantity of extracellular proteins.

No differences were shown between the PBS and 2% MetOH negative controls, in each of the microorganisms teste

			Concentration (mg/l)								
			**0 (PBS)**	**0 (2% MetOH)**	**0.001**	**0.01**	**0.1**	**1**	**10**	**100**	**1000**
E. coli	*B. cylindrica*	**λ (h)**	0.39±0.04	0.40±0.05	0.38±0.06	0.40±0.05	0.39±0.07	0.40±0.04	0.42±0.07	0.39±0.04	0.38±0.09
		**μm (A_{600}/h) × 10^{-3}**	1.42±0.04	1.41±0.03	1.40±0.02	1.45±0.05	1.39±0.06	1.41±0.07	1.38±0.06	1.40±0.03	1.38±0.06
		**YM ($_{600}$)**	0.65±0.05	0.63±0.06	0.64±0.04	0.70±0.05	0.66±0.03	0.73±0.08	0.66±0.06	0.61±0.05	0.72±0.04
		**R^2**	0.990±0.004	0.991±0.003	0.995±0.005	0.993±0.002	0.995±0.002	0.996±0.002	0.994±0.006	0.992±0.003	0.990±0.002
	L. racemosa	**λ (h)**	0.39±0.04	0.40±0.05	0.37±0.08	0.041±0.06	0.39±0.03	0.38±0.04	0.40±0.05	0.36±0.05	0.40±0.04
		**μm (A_{600}/h) × 10^{-3}**	1.42±0.04	1.41±0.03	1.45±0.04	1.39±0.05	1.38±0.06	1.41±0.02	1.42±0.03	1.42±0.02	1.37±0.06
		**YM ($_{600}$)**	0.65±0.05	0.63±0.06	0.65±0.04	0.66±0.03	0.60±0.07	0.57±0.08	0.57±0.07	0.67±0.05	0.58±0.04
		**R^2**	0.991±0.004	0.997±0.003	0.992±0.005	0.992±0.002	0.993±0.006	0.994±0.002	0.993±0.007	0.992±0.001	0.991±0.003
C. albicans	*B. cylindrica*	**λ (h)**	2.51±0.09	2.64±0.08	2.63±0.09	2.64±0.08	2.60±0.07	2.55±0.06	2.58±0.05	2.62±0.13	2.68±0.50
		**μm (A_{600}/h) × 10^{-3}**	7.47±0.52	7.51±0.32	7.60±0.08	7.62±0.39	7.63±0.41	7.54±0.17	7.51±0.22	7.60±0.22	6.51±0.17
		**YM ($_{600}$)**	0.52±0.06	0.50±0.01	0.49±0.02	0.49±0.02	0.49±0.03	0.45±0.03	0.44±0.01	0.46±0.06	0.47±0.06
		**R^2**	0.992±0.003	0.993±0.002	0.989±0.001	0.991±0.002	0.992±0.002	0.992±0.001	0.992±0.001	0.991±0.001	0.915±0.098
	L. racemosa	**λ (h)**	2.51±0.09	2.64±0.08	2.74±0.09	2.63±0.06	2.71±0.01	2.58±0.05	2.46±0.14	2.55±0.26	2.64±0.09
		**μm (A_{600}/h) × 10^{-3}**	7.47±0.52	7.51±0.32	7.18±0.26	7.19±0.31	7.56±0.36	6.92±0.13	6.91±0.14	6.97±0.36	7.31±0.70
		**YM ($_{600}$)**	0.52±0.06	0.50±0.01	0.54±0.02	0.51±0.02	0.49±0.07	0.45±0.03	0.45±0.04	0.46±0.02	0.47±0.03
		**R^2**	0.992±0.003	0.993±0.002	0.992±0.001	0.989±0.001	0.991±0.001	0.990±0.001	0.989±0.001	0.980±0.006	0.992±0.001
F. oxysporum	*B. cylindrica*	**λ (dy)**	1.17±0.07	1.14±0.05	1.19±0.04	1.10±0.03	1.18±0.10	1.18±0.07	1.15±0.12	1.11±0.16	1.20±0.04
		**μm (mm/day)**	4.72±0.10	4.71±0.02	4.80±0.04	4.70±0.07	4.84±0.06	4.82±0.03	4.71±0.14	4.66±0.12	4.78±0.20
		**YM (mm)**	55.07±1.37	55.07±4.24	53.53±3.21	55.77±1.52	53.50±3.82	58.19±1.19	55.74±1.41	56.72±4.95	55.67±1.83
		**R^2**	0.994±0.001	0.992±0.002	0.995±0.001	0.992±0.001	0.994±0.001	0.993±0.001	0.992±0.001	0.993±0.001	0.993±0.001
	L. racemosa	**λ (day)**	1.17±0.07	1.14±0.05	1.12±0.04	1.18±0.02	1.10±0.08	1.10±0.08	1.12±0.09	1.11±0.01	1.20±0.06
		**μm (mm/day)**	4.72±0.10	4.71±0.02	4.65±0.06	4.77±0.07	4.73±0.10	4.71±0.04	4.76±0.17	4.68±0.15	4.59±0.15
		**YM (mm)**	55.07±1.37	55.07±4.24	52.04±1.27	55.18±2.02	55.14±1.61	55.47±1.25	54.12±3.51	52.90±0.20	53.54±0.49
		**R^2**	0.994±0.001	0.992±0.002	0.9940.002±	0.997±0.001	0.994±0.001	0.994±0.001	0.994±0.002	0.995±0.001	0.996±0.001

Table S1. *E. coli*, *C. albicans* and *F. oxysporum* kinetic parameters obtained by interpolating microbial growth with the Gompertz model: lag time length (*λ*), maximum specific growth rate (μm), maximum growth (YM) and the Goodness of Fit (R^2). Data represent the mean ± standard deviation of three independent measurements. No statistically significant differences (Tukey's HSD, $p \leq 0.001$) were found among the values and the 2% MetOH negative controls.

	No. adhered cells or conidia/cm^2					
	E. coli		*C. albicans*		*F. oxysporum*	
**Conc. (mg/l)**	***B. cylindrica*** **(×10^8)**	***L. racemosa*** **(×10^8)**	***B. cylindrica*** **(×10^5)**	***L. racemosa*** **(×10^5)**	***B. cylindrica*** **(×10^5)**	***L. racemosa*** **(×10^5)**
**0 (PBS)**	8.22±2.24 a	8.22±2.24 a	2.50±0.23 a	2.50±0.23 a	7.2±0.7 a	7.2±0.7 a
**0 (2% MetOH)**	8.48±2.02 a	8.48±2.02 a	2.49±0.17 a	2.49±0.17 a	7.0±0.6 a	7.0±0.6 a
**0.001**	7.61±1.49 a	7.43±0.72 a	1.96±0.32 bc* (-21.4±3.5)	2.30±0.33 a*	7.4±0.8 a*	5.0±0.8 b* (-28.7±4.6)
**0.01**	7.97±2.04 a	7.89±0.61 a	1.87±0.53 c (-25.1±7.1)	2.14±0.31 a	5.9±1.1 a	5.5±1.1 b (-20.5±4.1)
**0.1**	8.21±2.22 a	8.28±1.71 a	1.92±0.56 cb (-23.1±6.7)	2.17±0.50 a	6.4±0.9 a*	5.3±0.9 b* (-24.3±4.1)
**1**	8.69±1.41 a*	3.79±0.35 b* (-55.4±5.1)	1.85±0.37 c* (-25.8±5.2)	2.19±0.33 a*	5.4±1.2 b (-23.0±5.2)	5.5±1.1 b (-21.6±4.3)
**10**	16.09±4.52 b* (+89.7±25.2)	3.86±1.53 b* (-54.5±21.7)	1.55±0.37 c* (-37.9±9.0)	1.82±0.33 b* (-26.9±4.9)	4.9±1.2 b (-28.9±6.7)	4.6±1.0 b (-34.0±7.5)
**100**	19.27±2.51 b* (+2.3±0.2 fold)	3.51±0.79 b* (-58.6±13.2)	0.87±0.18 d* (-64.9±13.5)	0.36±0.11 c* (-85.4±26.1)	5.1±2.1 b* (-27.0±10.9)	1.3±0.4 c* (-81.1±25.0)
**1000**	43.31±7.04 c* (+5.1±0.6 fold)	1.81±0.43 c* (-78.7±18.8)	2.33±0.58 ab* (-6.4±1.6)	0.58±0.17 c* (-76.8±22.9)	2.7±1.0 c* (-61.1±21.9)	1.6±0.5 c* (-76.3±23.1)

Table S2. Number of cells or conidia/cm^2 in the presence of *B. cylindrica* and *L. racemosa*. Different letters indicate statistically significant differences (Tukey's HSD, $p \leq 0.01$) among concentrations, whereas an asterisk implies a significant difference in the number of adhered cells or conidia/cm^2 between *B. cylindrica* and *L. racemosa* at the same concentration. In the brackets, percentage reduction/increase in comparison to 2% MetOH control sample is reported when significant. Data represent the mean ± standard deviation of at least three independent measurements.

	Biomass (μg proteins/cm²)					
	E. coli		*C. albicans*		*F. oxysporum*	
**Conc. (mg/l)**	***B. cylindrica***	***L. racemosa***	***B. cylindrica***	***L. racemosa***	***B. cylindrica***	***L. racemosa***
**0 (PBS)**	4.77±0.54 a	4.77±0.54 ab	0.65±0.05 a	0.65±0.05 a	6,05±0,11 a	6,05±0,11 a
**0 (2% MetOH)**	5.03±0.22 a	5.03±0.22 a	0.69±0.05 a	0.69±0.05 a	6,25±0,60 ab	6,25±0,60 ab
**10**	3.71±0.63 b* (-26.4±4.5)	4.68±0.55 ac*	0.80±0.10 ab*	0.40±0.08 b* (-41.6±8.0)	6,38±0,11 ab	6,22±0,18 a
**100**	3.70±0.50 b* (-26.4±3.6)	4.89±0.96 a*	0.90±0.09 b* (+30.1±3.0)	0.23±0.03 c* (-66.9±8.7)	6,04±0,04 a	5,38±0,15 b* (-13.9±0.4)
**1000**	3.58±0.11 b (-28.8±0.8)	3.81±0.56 bc (-24.3±3.6)	1.75±0.22 c* (+2.5±0.2 fold)	0.17±0.01 c* (-75.2±3.3)	6,74±0,22 b	2,63±0,19 c* (-57.9±4.1)

Table S3. Cellular biomass within the biofilm in the presence of *B. cylindrica* and L. *racemosa* in the presence of each extract. Different letters indicate statistically significant differences (Tukey's HSD, $p \leq 0.01$) among concentrations, whereas an asterisk implies a significant difference in the cellular biomass between *B. cylindrica* and *L. racemosa* at the same concentration. In the brackets, percentage reduction/increase in comparison to 2% MetOH control sample is reported when significant. Data represent the mean ± standard deviation of at least three independent measurements.

	EPS polysaccharides + proteins (µg/cm²)					
	E. coli		*C. albicans*		*F. oxysporum*	
**Conc. (mg/l)**	*B. cylindrica*	*L. racemosa*	*B. cylindrica*	*L. racemosa*	*B. cylindrica*	*L. racemosa*
**0 (PBS)**	1.90±0.20 a	1.90±0.20 a	2.96±0.58 a	2.96±0.58 a	9.11±1.28 a	9.11±1.28 a
**0 (2% MetOH)**	1.86±0.30 a	1.86±0.30 a	2.96±0.31 a	2.96±0.31 a	8.72±1.47 a	8.72±1.47 a
**10**	2.06±0.19 a	1.92±0.31 a	2.77±0.36 a	2.47±0.27 a	9.65±1.15 a	9.81±1.00 ab
**100**	2.40±0.57 a	2.00±0.19 a	2.75±0.26 a	2.44±0.32 a	13.57±1.83 b (55.7±7.5)	12.54±0.83 b (43.8±2.9)
**1000**	4.69±0.79 b (+2.5±1.2 fold)	4.60±1.07 b (+2.5±1.3 fold)	3.34±0.64 a*	4.65±0.99 b* (+56.9±12.1)	19.86±2.14 c* (+2.3±1.1 fold)	17.32±2.76 c* (98.6±15.7)

	EPS polysaccharides/proteins ratio					
	E. coli		*C. albicans*		*F. oxysporum*	
**Conc. (mg/l)**	*B. cylindrica*	*L. racemosa*	*B. cylindrica*	*L. racemosa*	*B. cylindrica*	*L. racemosa*
**0 (PBS)**	1.14±0.25 a	1.14±0.25 a	4.51±1.41 a	4.51±1.41 a	20.22±2.92 a	20.22±2.92 a
**0 (2% MetOH)**	1.09±0.35 a	1.09±0.35 a	4.39±0.93 a	4.39±0.93 a	18.34±3.04 a	18.34±3.04 a
**10**	1.16±0.21 a	1.24±0.39 a	8.32±3.17 a	6.18±1.39 a	20.92±2.57 a*	181.68±18.99 b* (+9.9±1.9 fold)
**100**	0.94±0.45 a	1.15±0.21 a	7.74±1.73 a	5.81±1.91 a	315.06±43.95 b* (+17.2±3.3 fold)	144.95±9.81 c* (+7.9±1.5 fold)
**1000**	2.48±0.72 b (+2.3±1.3 fold)	2.83±1.21 b (+2.6±1.6 fold)	9.61±3.37 b (+2.2±1.4 fold)	10.80±4.00 b (+2.5±1.5 fold)	923.97±111.27 c* (+50.4±6.9 fold)	229.42±37.26 d* (+12.5±1.9 fold)

Table S4. Extracellular polysaccharides and proteins sum and ratio in the presence of each extract. Different letters indicate statistically significant differences (Tukey's HSD, $p \leq 0.01$) among concentrations, whereas an asterisk implies a significant difference in the values between *B. cylindrica* and *L. racemosa* at the same concentration. In the brackets, percentage reduction/increase in comparison to 2% MetOH control sample is reported when significant. Data represent the mean ± standard deviation of at least three independent measurements.

	Intracellular ROS (A.U./µg proteins/cm²)					
	E. coli		*C. albicans*		*F. oxysporum*	
**Conc. (mg/l)**	***B. cylindrica***	***L. racemosa***	***B. cylindrica***	***L. racemosa***	***B. cylindrica***	***L. racemosa***
**0 (PBS)**	140.88±14.99 a	140.88±14.99 a	295.31±40.04 a	295.31±40.04 a	127.46±15.94 a	127.46±15.94 a
**0 (2% MetOH)**	135.80±14.99 a	135.80±14.99 a	321.64±11.81 a	321.64±11.81 a	123.50±9.19 a	123.50±9.19 a
**10**	142.89±9.08 a*	212.91±47.89 a*	167.80±38.34 b* (-47.8±10.9)	383.4±56.0 a*	120.84±12.65 a*	230.65±16.37 b* (+86.8±6.2)
**100**	163.16±4.81 a*	765.48±178.16 b* (+5.6±1.1 fold)	146.44±17.64 b* (-54.5±6.6)	418.3±37.8 a*	245.56±40.81 b* (+20.2±0.6)	364.24±22.92 c* (+2.9±0.1 fold)
**1000**	577.39±40.70 b* (+4.2±0.2 fold)	1124.71±40.85 c* (+8.3±0.3 fold)	1226.82±66.9 c* (+3.8±0.1 fold)	2876.9±357.6 b* (+8.9±0.9 fold)	2636.45±136.94 c* (+4.2±0.2 fold)	586.06±37.76 d* (+4.7±0.2 fold)

	Extracellular ROS (A.U./µg proteins/cm²)					
	E. coli		*C. albicans*		*F. oxysporum*	
**Conc. (mg/l)**	***B. cylindrica***	***L. racemosa***	***B. cylindrica***	***L. racemosa***	***B. cylindrica***	***L. racemosa***
**0 (PBS)**	66.57±3.30 ab	66.57±3.30 a	21.02±1.46 a	21.02±1.46 a	1.97±0.56 a	1.97±0.56 a
**0 (2% MetOH)**	63.25±5.21 a	63.25±5.21 a	20.06±2.54 ab	20.06±2.54 a	1.91±0.44 a	1.91±0.44 a
**10**	77.60±8.25 bc* (+22.7±2.4)	88.29±3.35 b* (+39.6±1.5)	16.58±2.02 bc* (-17.4±2.1)	31.15±4.80 b* (+55.3±8.5)	5.01±1.73 b (+2.6±0.6 fold)	3.71±0.74 a
**100**	83.95±6.55 c* (+32.7±2.6)	114.77±5.81 c* (+81.5±4.1)	14.93±2.94 c* (-25.6±5.0)	61.69±5.68 c* (+3.0±0.2 fold)	6.06±1.37 b* (+3.1±0.4 fold)	11.39±2.65 b* (+5.9±1.3 fold)
**1000**	77.68±12.30 c* (+22.8±3.6)	234.31±12.38 d* (+3.7±0.1 fold)	7.67±1.59 d* (-61.8±12.8)	67.11±9.60 c* (+3.3±0.3 fold)	17.12±3.66 c* (+8.9±1.9 fold)	78.12±6.88 c* (+39.9±3.6 fold)

Table S5. ROS. Intra- and extra-cellular level of ROS within the biofilm in the presence of *B. cylindrica* and *L. racemosa* extracts. Different letters indicate statistically significant differences (Tukey's HSD, $p \leq 0.01$) among concentrations, whereas an asterisk implies a significant difference in the ROS levels between *B. cylindrica* and *L. racemosa* at the same concentration. In the brackets, percentage reduction/increase in comparison to 2% MetOH control sample is reported when statistically significant. Data represent the mean ± standard deviation of at least three independent measurements.

	Detachment index (%)					
	E. coli		*C. albicans*		*F. oxysporum*	
**Conc. (mg/l)**	***B. cylindrica***	***L. racemosa***	***B. cylindrica***	***L. racemosa***	***B. cylindrica***	***L. racemosa***
**0 (PBS)**	2.38±0.26 a	2.38±0.26 a	18.78±2.65 a	18.78±2.65 a	8.83±0.64 a	8.83±0.64 a
**0 (2% MetOH)**	2.35±0.21 a	2.35±0.21 a	20.12±2.41 a	20.12±2.41 a	8.21±1.06 a	8.21±1.06 a
**10**	15.01±0.79 b* (+6.4±0.3 fold)	23.56±2.62 b* (+10.0±1.0 fold)	20.78±1.86 a	20.19±2.86 a	7.68±1.11 a	8.26±1.00 a
**100**	33.14±4.79 c* (+14.1±1.9 fold)	24.33±3.78 b* (+10.4±1.4 fold)	30.21±3.45 b* (+50.2±57)	19.82±1.54 a*	7.93±0.43 a*	11.62±1.60 a*
**1000**	29.95±6.41 d* (+12.8±2.5 fold)	21.09±2.63 b* (+9.0±1.0 fold)	35.44±0.70 c* (+76.1±1.5)	12.52±2.99 b* (-37.8±9.0)	4.03±0.34 b* (-50.9±4.3)	63.49±5.26 b* (+7.7±0.5 fold)

Table S6. Detachment indexes of biofilm pre-grown without extract and subsequently treated with *B. cylindrica* and *L. racemosa*. Different letters indicate statistically significant differences (Tukey's HSD, $p \leq 0.01$) among concentrations, whereas an asterisk implies a significant difference in detachment index between *B. cylindrica* and *L. racemosa* at the same concentration. In the brackets, percentage reduction/increase in comparison to 2% MetOH control sample is reported when statistically significant. Data represent the mean ± standard deviation of at least three independent measurements.
